# Early phenology and growth trait variation in closely related European pine species

**DOI:** 10.1002/ece3.3690

**Published:** 2017-12-03

**Authors:** Witold Wachowiak, Annika Perry, Kevin Donnelly, Stephen Cavers

**Affiliations:** ^1^ Centre for Ecology and Hydrology Edinburgh Penicuik Midlothian UK; ^2^ Institute of Dendrology Polish Academy of Sciences Kórnik Poland

**Keywords:** adaptive variation, common garden trial, quantitative traits, speciation

## Abstract

Closely related taxa occupying different environments are valuable systems for studying evolution. In this study, we examined differences in early phenology (bud set, bud burst) and early growth in a common garden trial of closely related pine species: *Pinus sylvestris, P. mugo,* and *P. uncinata*. Seeds for the trial were sourced from populations across the ranges of each species in Europe. Over first 4 years of development, clear differences were observed between species, while the most significant intraspecific differentiation was observed among plants from *P. sylvestris* populations from continental European locations. Trait differences within *P. sylvestris* were highly correlated with altitude and latitude of the site of origin. Meanwhile, *P. mugo* populations from the Carpathians had the earliest bud set and bud flush compared to other populations of the species. Overall, populations from the *P. mugo* complex from heterogeneous mountain environments and *P. sylvestris* from the Scottish Highlands showed the highest within‐population variation for the focal traits. Although the three species have been shown to be genetically highly similar, this study reveals large differences in key adaptive traits both among and within species.

## 1 INTRODUCTION

Photoperiod and temperature are important environmental drivers of distribution and local adaptation (Gaston, 2009). In forest trees, annual growth cycles have evolved in response to growing season length variation, making trade‐offs between survival and growth (Petit & Hampe, 2006). Across a species’ range, many phenotypic traits in trees show steep clinal variation that is usually interpreted as strong evidence for a balance between spatially variable selection and migration (Savolainen, Pyhäjärvi, & Knürr, 2007). In highly outcrossing species such as pines, among‐population variation in quantitative traits is often accompanied by very low population structure at neutral genetic loci due to the homogenizing effect of genetic dispersal by pollen and seed (Aitken, Yeaman, Holliday, Wang, & Curtis‐McLane, 2008). This indicates that divergent selection is strong enough to maintain population differentiation even in the presence of substantial gene flow (Storz, 2002). Patterns of population differentiation, estimated from quantitative trait variation in samples from across environmental gradients, have been studied in many forest tree species including *Quercus petraea* (Ducousso, Guyon, & Kremer, 1996), *Populus tremula* (Luquez et al., 2007), *Betula pendula* (Viherä‐Aarnio, Häkkinen, Partanen, Luomajoki, & Koski, 2005), and *Pinus sylvestris* (Hurme, Repo, Savolainen, & Paakkonen, 1997; Salmela, Cavers, Cottrell, Iason, & Ennos, 2011). However, comparative common garden studies of multiple closely related tree species have rarely been undertaken and have never previously been conducted simultaneously for the species investigated here. Studying closely related species in this way has the potential to improve our understanding of how the evolutionary process operates on adaptive trait variation across environmental gradients. Furthermore, such phenotypic data are essential to support genomic studies focussed on the genetic architecture of quantitative traits. Considering environmental change predictions, such studies are also important for assessment of mitigation strategies such as assisted migration or marker‐assisted breeding, which have been proposed for sustainable management of forest ecosystems.

The three pine species investigated here, Scots pine (*Pinus sylvestris* L.), dwarf mountain pine (*Pinus mugo* Turra *s.l*.), and mountain pine (*Pinus uncinata* Ramond), differ significantly from each other in phenotype, geographic range, and ecology. The species are phylogenetically closely related, and they are not reproductively isolated and diverged within the last 5 Myr (Wachowiak, Palme, & Savolainen, 2011). Taxonomically, they are treated as independent species or, excluding *P. sylvestris*, as subspecies in the *P. mugo* Turra complex (Businsky & Kirschner, 2006; Christensen, 1987a,b). Currently, they have largely disjunct ranges, following postglacial migration.

*Pinus sylvestris* is a tree of up to 40 m height and is socially, ecologically, and economically one of the most important forest tree species in the world. It has the largest range of all pines with populations from western Scotland to eastern Siberia and southern Spain to northern Scandinavia. *Pinus mugo* grows to a height of about 3 m, and it forms large, shrubby forests above the tree line up to an altitude of about 2,700 m with major populations in mountainous regions of western Europe (the Alps), central and eastern Europe (Sudetes, Carpathians), and southeast through Bosnia and Herzegovina, Montenegro, Serbia, and Romania to the Rila and Pirin mountains of Bulgaria, as well as several outlier populations (Critchfield & Little, 1966). *Pinus uncinata* is a tree of up to 20–25 m in height found in mountainous regions at altitudes of 1,400–2,700 m in the Pyrenees, the Massif Central, Western Alps as well as several marginal locations in the Iberian Peninsula (Hamerník & Musil, 2007).

Morphological and ecological divergence among these taxa is probably due to adaptation to their different habitats, enforced by the disjunction of their ranges and isolation in different Pleistocene refugia during glacial maxima (Christensen, 1987a,b). The species can be artificially crossed, and they hybridize in contact zones (Jasińska et al., 2010; Wachowiak & Prus‐Głowacki, 2008). Given that they currently occupy largely disjunct geographic ranges in ecologically distinct habitats, local adaptation has likely played a major role in their phenotypic diversification.

Although these species are clearly differently adapted and can be recognized based on growth form and size, previous studies have shown that they share a highly similar genetic background. For instance, they each have the same number of chromosomes (2*n* = 24) and share much ancestral genetic variation at common loci (Wachowiak, Boratyńska, & Cavers, 2013; Wachowiak, Palme, et al., 2011; Wachowiak, Salmela, et al., 2011). Net genetic divergence between the taxa is low (<0.1%), and no fixed differences have been found between species at nuclear gene loci (Wachowiak et al., 2013). Given this genetic background, we expect that the genetic architecture, and the set of underlying genes, governing adaptive trait variation within and among species should be similar when considered across similar environmental gradients. Despite their disjunct ranges, the species overlap to some extent in altitude, temperature, and rainfall range. Here, we might expect patterns of adaptive phenotypic variation to be similar in each species. Alternatively, adaptation to specific environmental variables may have involved selection for species–specific or high‐frequency divergent alleles. In this case, levels of intraspecific variation for the traits examined would be expected to differ between taxa.

In this study, we looked at patterns of variation in adaptively important quantitative traits across the geographic distribution of the species in Europe. Phenology (bud set, bud burst) and growth (height) data were collected over a 4‐year period from a progeny trial of young pines representing 28 populations across three species. We analyzed within‐ and among‐species variation to assess the strength of local adaptation at different ecological and evolutionary scales. We examined the extent of phenotypic differentiation in the traits at intra‐ and interspecific level. Specifically, we asked what is the amount of phenotypic variation within species considering their similar genetic background but contrasting distribution ranges.

## 2 MATERIAL AND METHODS

### 2.1 Sampling

**Figure 1:**
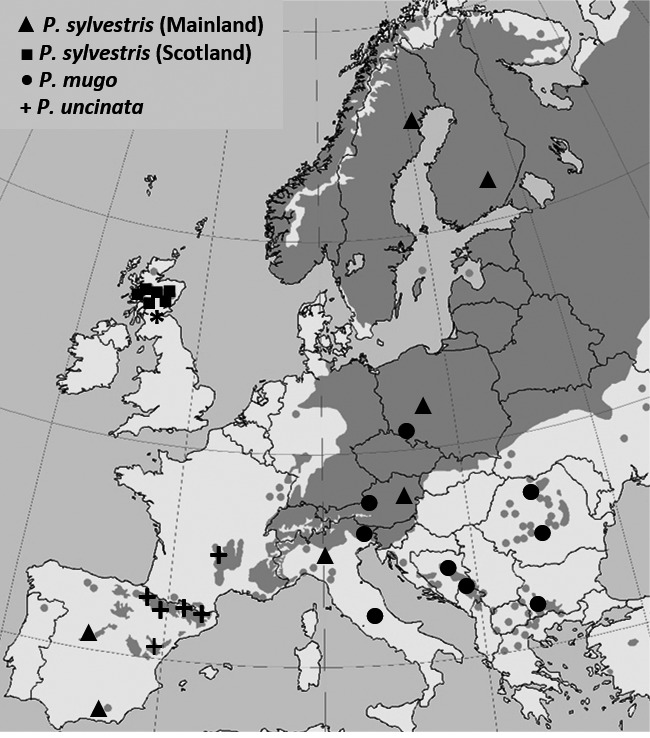
Geographic location of the sampled pine populations across Europe

Open‐pollinated seeds of the three pine species were collected from twenty‐eight natural populations in Europe covering the extent of each species range, including thirteen populations of *P. sylvestris*, nine *P. mugo,* and six *P. uncinata* (Table 1, Figure 1). Cones were collected from five mature trees from each population. Trees were separated by at least 50 m to minimize sampling of closely related individuals. Furthermore, the trees were selected to represent typical stands from each location avoiding sampling from population margins or extreme habitats within each stand especially in the mountain areas. Species and populations were sampled across environmental gradients in latitude (temperature, photoperiod), altitude (mountain, lowland), and growing season length to maximize the environmental amplitude that has given rise to adaptive responses in phenology and growth. Trees need to balance growth and dormancy periods to successfully compete for resources within populations while avoiding frost damage. We focused on these traits as they are under strong selective pressure in natural populations.

**Table 1:** Geographic location, altitude, and mean annual temperature for the sampled pine species populations from Europe

Acr.	Population	*N*	Long.	Lat.	Alt. (m)	Temp. (°C)
S1	Sweden, Tjärnbergshedens	5	20.74	64.61	110	1.28
S2	Finland, Punkaharju	5	29.36	61.73	80	3
S3	Poland, Jarocin	5	17.48	51.97	120	8.2
S4	Austria, Pernitz	5	16.00	47.91	500	9.1
S5	Italy, Cella di Palmia	5	10.17	44.63	340	12.5
S6	Spain, Trevenque	5	−3.55	37.10	1,170	15.7
S7	Spain, Valsain	3	−4.04	40.87	1,350	11.3
S8	SCO, Shieldaig	3	−5.64	57.51	81	7.6
S9	SCO, Glen Tanar	5	−2.86	57.06	160	7.8
S10	SCO, Rothiemurchus	5	−3.77	57.15	318	6.5
S11	SCO, Glen Affric	5	−4.92	57.27	256	7.65
S12	SCO, Black Wood of Rannoch	5	−4.32	56.67	275	7.65
S13	SCO, Glen Loy	5	−5.13	56.91	170	7.65
M1	Poland, Śląskie Kamienie	4	15.60	50.78	1,300	0.4
M2	Austria, Karwendel Alps	5	11.30	47.38	1,400	8.7
M3	Italy, Karnische Alps	5	13.26	46.55	1,530	7.5
M4	Italy, Abruzzi Mts.	5	14.08	42.08	2,200	13.9
M5	Romania, Eastern Carpathians	4	24.80	47.57	1,720	7.9
M6	Romania, Southern Carpathians	4	25.45	45.43	2,070	−2.6
M7	BH, Belashnica Mts.	3	18.22	43.75	2,120	9.5
M8	Montenegro, Durmitor Mts.	5	19.09	43.16	2,100	6.9
M9	Bulgaria, Pirin Mts.	3	23.42	41.77	2,000	13.9
U1	France, Col de la Croix de Morand	5	3.05	45.68	1,400	10.9
U2	Andora, Pyrenees, Vall de Ransol	5	1.59	42.63	2,025	13.4
U3	Andora, San Miguel de Engolasters	5	1.57	42.52	2,000	13.4
U4	Spain, Castiello de Jaca	5	−0.54	42.64	1,720	−1.3
U5	Spain, Sierra de Gudar	5	−0.61	40.39	2,000	11.5
U6	Spain, Pyrenees, Benasque	5	0.66	42.69	2,000	−1.2

S, *Pinus sylvestris*; M, *P. mugo*; U, *P. uncinata*; SCO, Scotland; BH, Bosnia and Herzegovina; N, Number of families studied from each location.

### 2.2 Progeny trial experiments

Genetic variation in phenotypic traits can be identified by growing young pines in a common garden environment in which environmental variation is kept to minimum (e.g., White, Adams, & Neale, 2007). Seeds from common mother trees were sown on trays (75:25 compost type John Innes #1: sand) in spring 2010. After germination, a provenance–progeny trial was established in an unheated glasshouse at the Centre for Ecology and Hydrology, Edinburgh, UK. Seedlings were transferred to 0.62 l pots (diameter 11 cm, depth 9.6 cm) and grown under natural light conditions (glasshouse was shaded to avoid excess light) with automatic watering applied during the growing season. The glasshouse temperature was set to track outside temperature ±3°C, and watering was automatic at 4 times a day for 3 min 30 s—typically, this was sufficient to keep pots moist; however, a control pot containing a soil humidity probe was used to regulate a minimum soil moisture (70%), below which additional watering would be applied. For most populations, the trial consisted of five families/population and the trial was divided into 18 randomized blocks (Table 1), with a single seedling per family per block. In total, 1623 young pines were analyzed including 392 from *P. sylvestris* (continental), 365 from *P. sylvestris* (Scotland), 399 from *P. mugo,* and 467 from *P. uncinata*. The average 2010–2011, 2011–2012, and 2012–2013 winter temperatures (October–March) were 4.1°C, 3.8°C, and 4.0°C, respectively. The lowest glasshouse temperature recorded was −2.6°C (February 2012). Mean temperature for the growing season across all years was 12–15°C.

### 2.3 Quantitative trait data analysis

Phenology (timing of bud set/ bud burst) and total height were recorded for every seedling to evaluate within‐ and between‐species variation. Bud set was measured from August to October 2010 as the number of days since the first scoring date (i.e., since the date on which the first plant that set terminal bud was observed). Bud set was scored when a visible apical bud with clearly developed scales was formed at the tip of a stem in each seedling. Bud burst was scored as emergence of the new needles around the tip of the apical bud in the main stem. It was measured during spring 2011 and 2012 starting from the day when the first bud burst was observed. Phenology observations were conducted twice a week. The height of young pines was measured in December 2011, 2012, and 2013. As the numbers of populations and families within species varied, we employed statistical models that were robust to unbalanced designs. Unbalanced analysis of variance (families nested within populations) was used to test for between species, population, and family differences in phenology and growth performance. A group of six Scottish populations of *P. sylvestris* were analyzed separately as they cross a strong west–east environmental gradient at a narrow spatial scale (Salmela et al., 2010). This approach allowed us to evaluate adaptation at a local scale without introducing bias in the analysis of populations across the full range due to overrepresentation of Scottish populations. Populations were considered fixed factors, and families within populations and blocks were random. Variance components due to populations, families, and blocks were estimated using a mixed‐model (REML) approach. Similarly, interspecies comparisons were conducted treating species as a fixed factor. Analyses were carried out using GenStat 13.1.0.4470.

### 2.4 Environmental covariance

We investigated whether differences in climatic conditions have induced phenotypic adaptive variation among natural populations by looking at trait variation associated with altitude and latitude. As variation in the traits analyzed is related strongly to location, and because geographic descriptors capture unmeasured environmental factors that might also be selectively important, we used latitude and altitude as predictors for the investigated traits. Data on mean temperature, which affects species phenology and growth, were also extracted from Met Office and WorldClim databases (Table 1), although, in general, values of mean temperature were correlated with the geographic descriptors of each site (Table S1).

Mixed‐model analyses were performed in *R* (www.R-project.org/), using a methodology similar to that described by Grueber, Nakagawa, Laws, and Jamieson (2011). For each trait, a global linear mixed‐effects model was first specified using the package “lme4” (Bates, Mächler, Bolker, & Walker, 2015) as follows:

Trait=μ+Latitude+Altitude+Population+Family+Block+ε

where the covariates latitude and altitude (m) at sites of origin were fixed effects; population, family within population, and block were treated as random effects with random intercepts. After model fitting, covariates were rescaled to a mean of zero and a standard deviation of 0.5 using the package “arm” (Gelman & Su, 2014). Full model subsets were generated and evaluated by AICc using the package “MuMIn” (Barton, 2015) (R script is provided in Appendix S1). In addition, for each species/trait, a random‐effects model was specified to evaluate the contribution of population, family within population, and block to the total variance in the absence of additional covariates. Covariation between selected pairs of traits was examined in a similar manner as described for environmental covariates. The first of each trait pair was defined as the dependent variable; models with and without the second trait as a covariate were then compared in terms of AICc. Covariates were retained on their original scales. Population, family within population, and block were fitted as random effects.

## 3 RESULTS

### 3.1 Phenotypic differentiation between species

**Figure 2:**
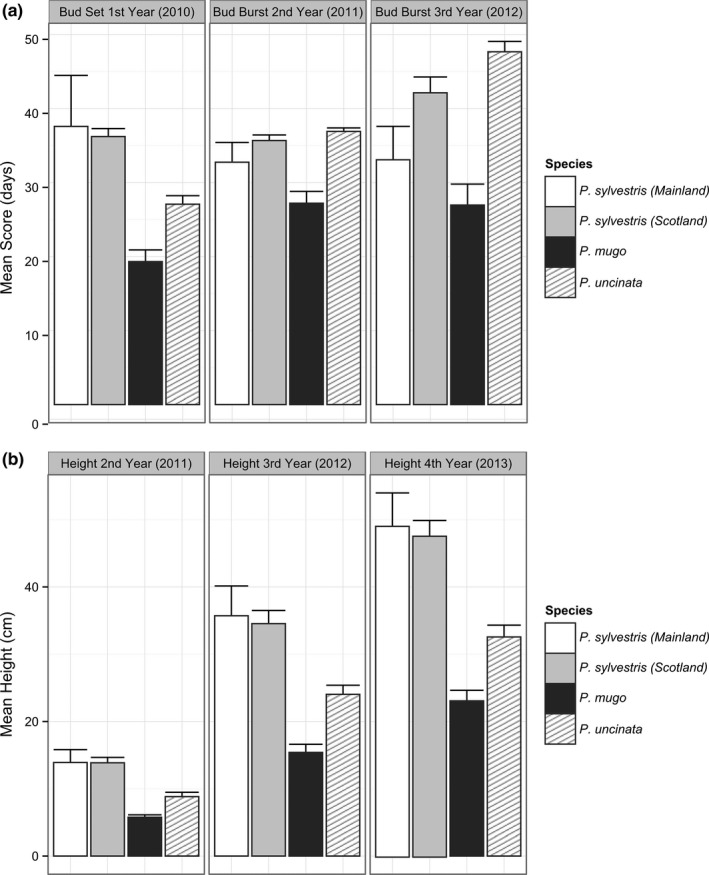
Phenological (bud set and bud burst, a) and growth (height, b) variation between pine species and their standard errors

**Figure 3:**
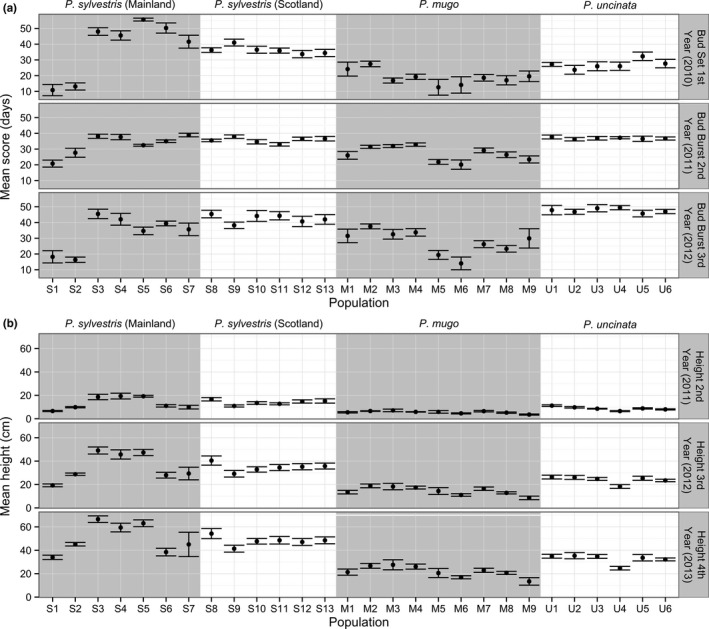
Bud set and bud burst (a) and growth (height) (b) variation between populations and their standard errors (Table 1)

There was a significant difference (*p* < .0016 for all traits) between species in phenology and growth (Figure 2, Table S2). On average, the mountain species, *P. mugo,* had the earliest bud set; however, this still occurred later than the most northern populations of *P. sylvestris* (Figure 3, Table S3). Populations of *P. sylvestris* from northern Sweden set terminal buds earlier than any other population across all species (Figure 3). Most of the *P. sylvestris* populations set terminal buds later than other species. *Pinus mugo* was the first to terminate dormancy and had the slowest growth (Figure 2). Bud burst occurred latest in *P. uncinata*; however, it was similar to some *P. sylvestris* populations especially from central Europe and Scotland. The fastest growing individuals belonged to *P. sylvestris* populations from Poland, Austria, and Italy (Figure 3, Table S3). Population means for the timing of first‐year (2010) bud set ranged over 44 days in *P. sylvestris*, 14 days in *P. mugo,* and 8 days in *P. uncinata*.

### 3.2 Phenotypic differentiation within species

There was significant between‐population variation in phenology among *P. sylvestris* from mainland locations and among *P. mugo* populations, but not among Scottish *P. sylvestris* populations or among *P. uncinata* populations (Table S4). Continental *P. sylvestris* and *P. mugo* had large ranges of variation among population means for bud flush (from about two to 4 weeks between first and last scores) (Figure 3, Table S3).

**Figure 4:**
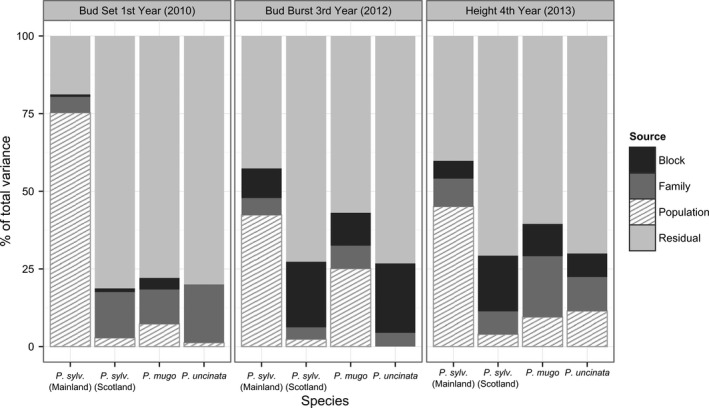
Variance components for phenology and growth in the three pine species

For *P. sylvestris*, the largest difference in phenology was observed between northern European populations (Finland and Sweden) and all other locations (Figure 3). In general, populations from northern Europe set bud and flushed earlier than southern populations. For example, northern populations of *P. sylvestris* set terminal buds from about three to 6 weeks earlier than Scottish and southern European populations, respectively. The average heights of *P. sylvestris* populations from the range margins in Spain and northern Europe were around 40% smaller than continental populations from central Europe. The largest differences in height were observed between populations from Poland and Sweden (the fastest and slowest growing populations, respectively). Populations from Scotland exhibited intermediate heights. No significant differences were observed among Scottish populations for any of the traits considered (Table S4). In mainland *P. sylvestris* populations, differentiation between populations was the major component of variation for all traits throughout the experiment (Figure 4, Table S5).

In *P. mugo*, populations from the Carpathians set terminal buds earlier than other populations. Differences in bud burst were observed along a longitudinal cline between populations from the Sudetes Mountains and Alps to the Carpathians and Balkan region. In general, more rapid growth was observed in *P. mugo* from the western part of the species range in populations from the Alps and Abruzzi Mts. The slowest growing individuals were from the most southern populations from the Pirin Mountains, Bulgaria. Differentiation between *P. mugo* populations was only the major component of variation for bud burst. There was a large between family component of variation for other traits in this species (Figure 4, Table S5).

*Pinus uncinata* was the most phenotypically homogenous and did not show any significant variation between populations for phenological traits (Table S4). Significant between‐population differences in height in this species were driven by one population from Spain (Castiello de Jaca) that showed the slowest growth compared to other populations.

Large differences between families within populations were observed for bud set in *P. sylvestris* and *P. uncinata* and for bud burst in *P. uncinata* in the second year of measurements (Table S4). There was significant difference in height between families within populations in each species in the second year of measurements (Table S4).

### 3.3 Environmental associations and covariation between traits

Overall, the populations of origin of our *P. sylvestris* plants were distributed across a much larger latitudinal range and at lower elevations than the taxa from the *P. mugo* complex (Figure S1), and altitude and latitude were highly negatively correlated (*r* ^2^ = .82, *p* < .01) (Table S1). However, for continental populations of *P. sylvestris*, evidence was found for an effect of altitude and latitude of population origin on both bud set and height (Table 2). For phenological traits, latitude had an effect size approximately twice that of altitude, whereas for height the effect sizes were very similar. In the third year of bud burst measurements, effects of altitude and latitude at population origin were found in *P. mugo*. In this case, the effect sizes of altitude and latitude were similar. No environmental associations were observed for phenotypic trait variation among populations of *P. uncinata* or Scottish populations of *P. sylvestris*.

**Table 2:** Effects of environmental variables (altitude and latitude; please note that the coefficients are standardized) on phenotypic variation based on mixed‐model analysis (REML). ΔAICc values represent the difference in AICc between the null model (no fixed effects) and the best (a value of zero indicates that the null model was the best); Akaike weight represents the probability of that model being the best out of the set considered; *df*, degrees of freedom

Trait	Species	Intercept (*SE*)	Altitude (*SE*)	Latitude (*SE*)	*df*	ΔAICc	Akaike weight
Bud set first year (2010)	*P. sylvestris* (mainland)	42.31 (1.52)	−25.38 (4.4)	−46.56 (4.35)	7	20.71	1.00
	*P. sylvestris* (Scotland)	36.29 (0.99)	–	2.59 (1.88)	6	0.21	0.36
	*P. mugo*	19.47 (1.49)	−4.19 (2.83)	–	6	0.32	0.31
	*P. uncinata*	27.07 (1.15)	–	–	5	0.00	0.37
Bud burst second year (2011)	*P. sylvestris* (mainland)	33.38 (1.85)	–	−8.96 (3.13)	6	4.74	0.59
	*P. sylvestris* (Scotland)	35.52 (0.67)	−1.77 (1.22)	−1.81 (1.19)	7	0.16	0.31
	*P. mugo*	27.20 (1.59)	–	–	5	0.00	0.36
	*P. uncinata*	36.90 (0.47)	–	–	5	0.00	0.43
Bud burst third year (2012)	*P. sylvestris* (mainland)	35.73 (3.37)	−13.5 (8.99)	−24.75 (9.07)	7	3.97	0.54
	*P. sylvestris* (Scotland)	42.12 (2.14)	–	–	5	0.00	0.50
	*P. mugo*	28.71 (2.04)	−25.91 (7.14)	−20.13 (6.22)	7	5.79	0.90
	*P. uncinata*	47.66 (1.40)	–	–	5	0.00	0.46
Height second year (2011)	*P. sylvestris* (mainland)	15.35 (1.11)	−12.75 (3.18)	−13.32 (3.16)	7	7.79	0.96
	*P. sylvestris* (Scotland)	13.86 (0.81)	–	–	5	0.00	0.49
	*P. mugo*	5.80 (0.34)	−0.94 (0.68)	–	6	0.17	0.31
	*P. uncinata*	8.83 (0.65)	–	–	5	0.00	0.34
Height third year (2012)	*P. sylvestris* (mainland)	39.15 (2.45)	−29.43 (6.24)	−27.96 (6.24)	7	8.83	0.98
	*P. sylvestris* (Scotland)	34.55 (1.95)	–	–	5	0.00	0.48
	*P. mugo*	15.42 (1.20)	–	–	5	0.00	0.33
	*P. uncinata*	24.04 (1.35)	–	–	5	0.00	0.51
Height fourth year (2013)	*P. sylvestris* (mainland)	53.69 (2.61)	−34.45 (7.08)	−30.17 (7.11)	7	9.06	1.00
	*P. sylvestris* (Scotland)	47.69 (2.19)	–	–	5	0.00	0.51
	*P. mugo*	23.09 (1.56)	–	–	5	0.00	0.34
	*P. uncinata*	32.57 (1.76)	–	–	5	0.00	0.48

For all species distributions, a negative relationship was observed between bud set 2010 and bud burst the following year; bud burst occurs earlier on average when bud set was later, although little evidence was found for this effect in *P. sylvestris* mainland populations (ΔAICc = 0.69, Table S6). Average height in 2011 was found to be lower in Scottish populations of *P. sylvestris* and in *P. mugo* for individuals which set bud later in 2010. Bud burst and height were found to be positively related in 2011 and 2012; however, the effect was not detected consistently across both years for all distributions (Table S6).

## 4 DISCUSSION

### 4.1 Phenotypic variation at interspecific level

The focal pine species diverged from a common ancestor about five million years ago (Wachowiak, Palme, et al., 2011). At present, they have mostly disjunct ranges and are adapted to different environmental gradients from northern to southern Europe that experience differing selective pressures in relation to temperature, photoperiod, and water availability. In our study, we grew individuals from seed sampled from populations located across the European range. Trial plants were raised under controlled environmental conditions in a glasshouse to assess variation in key phenotypic traits relevant to species evolution and intraspecific local adaptation. We focused on using geographic descriptors (latitude, altitude) to assess trait variation as these have strong relationships with variation in the phenology and growth traits studied. Common garden experiments are useful for studying genetic variation in phenotypic traits as they minimize variation in environmental conditions. In our case, glasshouse conditions did not reflect those at the home sites of any of the provenances studied and were effectively novel to all test plants; therefore, we expected expression of latent variation and a wider range of variability in phenotype that might be expected at their home sites. However, as conditions were common to all plants, any significant inter‐ or intraspecific differences observed in our study in the analyzed traits should have a strong heritable component. Although not specifically addressed by our experimental design, it cannot be excluded that some of the observed phenotypic differentiation between populations and species could be affected by epigenetic interactions. Such heritable changes in gene function that do not result explicitly from sequence variation may contribute to phenotypic plasticity and adaptation. However, the influence of epigenetics effects on phenotypic diversity is much less known in pines that may not be affected by epigenetic interaction in a similar fashion like other conifers, e.g., spruce species (Gömöry, Foffová, Longauer, & Krajmerová, 2015; Kohmann & Johnsen, 1994; Yakovlev, Fossdal, & Johnsen, 2010). Also, side effects of the maternal environment are at least partially accounted for by the family component in our models (Cendán, Sampedro, & Zas, 2013; Zas, Cendán, & Sampedro, 2013).

Our data showed that these closely related pine species have evolved significant differences in phenology and height. In general, *P. sylvestris* was phenotypically distinct from the other two species. However, there was some overlap in phenology between species. For instance, populations of *P. sylvestris* from Finland had similar bud set timing to *P. mugo* populations from the Carpathians, and *P. mugo* from Austria had similar bud set timing to *P. uncinata* populations. We also observed some similar patterns of covariation between traits in different species. Differences in height among populations were more consistent than differences in phenological traits. *Pinus sylvestris* showed more rapid growth than the species from the *P. mugo* complex. Reduced height can be advantageous in mountain regions due to the risk of damage from strong wind and avalanches. This is particularly true for *P. mugo,* which forms extensive shrubby populations at high altitudes above the tree line, and had the slowest growth compared to other species. The lower level of variation in phenology and growth between taxa from the *P. mugo* complex than between the latter and *P. sylvestris* corresponds to adaptation to similar and relatively narrow environmental niche occupied by the species from the *P. mugo* complex. These patterns are reflected in estimates of evolutionary divergence from biometric, biochemical, and molecular data (Boratynska, Jasinska, & Boratynski, 2015; Lewandowski, Boratyński, & Mejnartowicz, 2000). Despite differences in growth form, the taxa from the *P. mugo* complex have shown no diagnostic anatomical or morphological needle traits, or molecular markers (Boratynska et al., 2015; Jasińska et al., 2010; Wachowiak, Odrzykoski, Myczko, & Prus‐Głowacki, 2006). Therefore, some biometric markers often used in pine taxonomy could not be applied to verify, e.g., hybrid origin of trees from contact zones of the species (Boratynska et al., 2015).

### 4.2 Patterns of within‐ and between‐population differentiation

At the within‐species level, the taxa showed different patterns of within‐ and between‐population variations. In *P. sylvestris*, we found substantial between‐population variation that was highly correlated with latitude and/or altitude of population origin. In general, northern populations from Finland and Sweden set bud and flushed earlier than more southerly populations following general patterns in forest trees related to photoperiod and temperature differences (Hurme et al., 1997; Rohde, Bastien, & Boerjan, 2011). On the other hand, populations from Spain, Scotland, and northern Europe (Finland and Sweden) showed slower growth compared to central European (Poland and Austria) and Italian populations. In previous studies, significant phenotypic differentiation between distant populations of *P. sylvestris* was observed in timing of growth cessation and frost hardiness (Koski & Sievänen, 1985). When seedlings from different parts of Europe were grown under photoperiods typical of 50° latitude, significant differences were observed in the timing of bud set of young pines from northern versus southern regions (Oleksyn et al., 1992) and height growth cessation of older trees (Oleksyn, et al. 1998; Repo et al., 2000). Furthermore, provenance transfers of *P. sylvestris* from north to south of Europe resulted in increased survival but reduced growth as compared to local populations (Eriksson et al., 1980; Persson & Ståhl, 1990), supporting the observations of clinal variation of phenotypic traits in the species (Langlet, 1959). In contrast, no clear correlation with latitudinal clines was observed in our study for the two taxa from the *P. mugo* complex. Some evidence of between‐population differentiation in bud burst (third year of measurements) weakly associated with environmental gradients was found in *P. mugo*. The most southerly populations, from Italy, set terminal buds about 5 days earlier than the most northerly populations from Poland. The most homogenous species in our dataset was *P. uncinata*. Based on microsatellite loci (Dzialuk et al., 2009), populations of *P. uncinata* differ genetically across its range, presumably as a result of different postglacial histories. However, our data do not show any differences in phenology or height between *P. uncinata* populations. With the exception of one outlier population, which showed slower growth, there was no significant between‐population differentiation within *P. uncinata*.

In general, variation between families within populations was much more pronounced in the taxa from the *P. mugo* complex and *P. sylvestris* from Scotland compared with the mainland range of the latter. In our study *P. sylvestris* populations covered the largest latitudinal gradient and consisted of lower altitude populations in comparison with the species from the *P. mugo* complex. In general, we expect more homogenous environmental conditions within populations in lowland *P. sylvestris* stands than in mountain populations of the *P. mugo* complex in Europe or *P. sylvestris* populations from the Scottish Highlands. In our dataset, we observed between family differences in phenology and height that were much stronger in populations derived from mountainous regions. Significant among‐ and within‐ population variations have also been observed in a common garden trial of *P. sylvestris* sampled from the diverse environments of the Scottish Highlands (Salmela et al., 2011). In this experiment, samples were grown outdoors and experienced strong winter chilling conditions that differed from our glasshouse‐based experiment. High within‐population phenotypic diversity may result from the high spatial heterogeneity of the environment in these mountain regions, which could cause spatially variable selection pressure and/or variable efficiency of gene flow at fine spatial scales. High genetic diversity of the populations could also result from recent admixture of populations of different postglacial origin that maintained their phenotypic diversity. For instance, Scottish populations showed some unique mitochondrial genetic variation (Sinclair, Morman, & Ennos, 1998) and high genetic diversity as compared to mainland populations (Wachowiak, Salmela, et al., 2011)—patterns not compatible with a simple model of recolonization of the British Isles from continental Europe after last glacial maximum.

Between‐population variation within species has been observed in previous morphological and biochemical studies. Differences were detected between east and south Carpathian and other populations of *P. mugo* in a biometric study of needle and cone characters (Boratynska et al., 2015) and allozyme loci (Sannikov, Petrova, Schweingruber, Egorov, & Parpan, 2011). Differentiation was explained as being due to different Holocene histories and Pleistocene isolation that influenced genetic variation between those parts of the species range. Biometric data also indicated differentiation of *P. mugo* populations from the Balkans, represented in our dataset by the Pirin Mountain population. This was explained as being due to colonization of the Balkans from two different refugia in the Alps and southern Carpathians (Boratynska et al., 2015). However, those populations showed no evidence of significant genetic divergence at neutral markers when compared to putative source populations (Wachowiak et al., 2013). Some differences between Pyrenean and marginal populations of *P. uncinata* in the Massif Central and Spain were found in previous biometric and genetic studies (Boratynska et al., 2015; Dzialuk et al., 2009). These studies also found some evidence of morphological differences among Pyrenean populations, represented in our study by populations from Benasque and Castiello de Jaca versus San Miguel de Engolasters and Vall de Ransol. However, in our study, the only significantly differentiated population was from Castiello de Jaca, which showed slower growth than all other populations.

### 4.3 Underpinning future searches for genes of adaptive significance

Recent studies of nucleotide sequence variation showed high genetic similarity between the focal species: they share a high proportion of common, ancestral variation (Wachowiak et al., 2013; Wachowiak, Palme, et al., 2011). The combination of recent evolutionary divergence and substantial ecological differentiation means these species form a valuable model for studies of the genetic basis of local adaptation and speciation. Our data show that the mean and level of variation in important phenotypic traits differ between taxa. Given this evidence for quantitative phenotypic divergence, studies of variation at the genome level can now be better focussed on searches for genes that have experienced selection during the process of speciation. Correspondingly, linking analysis of within‐species phenotypic variation to analysis of patterns of molecular polymorphism will help to verify which genes are under selection due to adaptation to local environmental conditions. However, as many phenotypic traits are polygenic, searches for genes involved in local adaptation and speciation must involve many genomic regions. Recently developed genomic resources for *P. sylvestris* and the taxa from the *P. mugo* complex, comprising whole transcriptome sequences and a large database (over 200,000) of single nucleotide polymorphisms (SNPs), provide a valuable set of markers for such analysis (Wachowiak, Trivedi, Perry, & Cavers, 2015). Such studies should also address epigenetic variation that may be important in local adaptation for long‐lived organisms like forest trees, which experience substantial environmental variation during their life spans.

## 5 CONCLUSION

Our data demonstrate significant between‐species variation in phenology and height. Distinct intraspecific patterns of phenotypic variation indicate that selection has operated differently on traits important for adaptation to the environments occupied by individual species. Approaches for resolving the genetic architecture of adaptive traits in species derived from heterogeneous environments such as the taxa from the *P. mugo* complex should take into account the high within‐population variation of the studied traits. Comparison of genetic variation between populations could be effective in species like *P. sylvestris* which exhibit strong clines in adaptive traits, assuming that they represent populations of shared evolutionary and demographic history (Wachowiak et al. 2014). The study demonstrates genetically driven variation in pine phenotypes across the species distribution in Europe. This variation is important for population resilience, and better knowledge about its distribution and effects can advance management practice as forests are challenged by future environmental changes.

## CONFLICT OF INTEREST

None declared.

## AUTHOR CONTRIBUTIONS

WW and SC designed and planned the research; WW generated data and wrote the manuscript; WW and KD analyzed the data; AP contributed to acquisition of the data; all authors critically revised the manuscript for content and approved the final version.
