# Supplementary material for: Early phenology and growth trait variation in closely related European pine species
Source: Ecol Evol. 2017 Dec 3;8(1):655–66. doi: 10.1002/ece3.3690 (PMC5756864; doi:10.1002/ece3.3690)

Supporting information

Supplementary Table S1. Correlation for environmental parameters using Pearson's coefficient.

***P. sylvestris* (mainland)**				***P. sylvestris* (Scotland)**		
	**Longitude**	**Altitude**	**Temperature_mean**	**Longitude**	**Altitude**	**Temperature_mean**
**Latitude**	0.91	-0.82	-0.98	0.04	-0.28	-0.22
**Longitude**		-0.86	-0.89		0.36	-0.21
**Alt.**			0.72			-0.59
P. mugo				***P. uncinata***		
	**Longitude**	**Altitude**	**Temperature_mean**	**Longitude**	**Altitude**	**Temperature_mean**
**Latitude**	-0.19	-0.88	-0.64	0.86	-0.82	0.01
**Longitude**		0.44	-0.24		-0.86	0.28
**Altitude**			0.31			0.09
**All Populations**						
	**Longitude**	**Altitude**	**Temperature_mean**			
**Latitude**	-0.04	-0.82	-0.40			
**Longitude**		0.23	-0.17			
**Altitude**			0.11			

Supplementary Table S2. Output from ANOVA for phenology and height variation between species. Degrees of freedom: d.f.; mean sum of squares: m.s.; F: F value; p: significance value.

**Trait**	**d.f.**	**m.s.**	**F**	**p**
Bud set 1st year (2010)	2	53473.4	12.42348	0.0002
Bud burst 2nd year (2011)	2	6756.75	8.461591	0.0016
Bud burst 3rd year (2012)	2	34447.9	12.55436	0.0002
Height 2nd year (2011)	2	10990.5	23.33539	<.0001
Height 3rd year (2012)	2	58197.84	24.67181	<.0001
Height 4th year (2013)	2	95993.6	33.10923	<.0001

Supplementary Table S3. Population means (standard error) for bud set (in the unit of days since first record), bud burst (days since first record) and height (centimetres) in the three pine species.

Population	Bud set 1st year (2010)	Bud burst 2nd year (2011)	Bud burst 3rd year (2012)	Height 2nd year (2011)	Height 3rd year (2012)	Height 4th year (2013)
S1	10.8 (3.6)	20.8 (2.2)	18.2 (3.9)	6.6 (0.5)	19.3 (1.2)	34.0 (1.8)
S2	13.1 (2.3)	27.7 (2.9)	16.3 (1.7)	9.9 (0.5)	28.8 (1.0)	45.2 (1.5)
S3	48.0 (2.4)	38.1 (1.3)	45.5 (3.0)	18.6 (2.3)	49.1 (3.0)	66.6 (2.8)
S4	45.5 (3.0)	37.7 (1.7)	42.0 (3.7)	19.4 (2.5)	45.7 (3.9)	59.4 (3.7)
S5	55.6 (0.9)	32.4 (0.6)	34.7 (2.4)	19.2 (0.8)	47.4 (2.6)	63.1 (2.9)
S6	50.3 (3.2)	35.0 (0.8)	39.4 (1.5)	11.0 (1.0)	28.0 (2.5)	38.5 (3.3)
S7	41.6 (4.1)	38.9 (1.2)	35.7 (4.0)	9.9 (1.6)	29.4 (5.4)	45.0 (10.4)
S8	36.3 (1.5)	35.5 (0.8)	45.4 (2.4)	16.7 (1.4)	40.5 (3.9)	54.3 (4.3)
S9	41.0 (2.2)	37.9 (1.1)	38.2 (2.0)	10.9 (0.9)	29.2 (2.8)	41.4 (2.9)
S10	36.5 (2.2)	34.6 (1.4)	44.1 (3.5)	13.5 (1.1)	32.9 (2.3)	47.7 (2.4)
S11	35.9 (1.6)	33.0 (1.1)	44.3 (2.6)	12.8 (0.9)	34.5 (2.6)	48.6 (3.2)
S12	33.7 (2.3)	36.5 (1.1)	40.7 (3.3)	14.8 (1.4)	35.2 (2.7)	47.1 (2.9)
S13	34.4 (2.3)	36.6 (1.5)	42.0 (3.1)	15.2 (1.8)	35.8 (2.5)	48.5 (2.9)
M1	24.2 (4.4)	26.0 (2.4)	31.5 (4.3)	5.5 (0.6)	13.5 (1.5)	21.3 (2.6)
M2	27.4 (1.8)	31.4 (1.0)	37.5 (1.6)	6.6 (0.3)	18.8 (1.6)	26.7 (2.0)
M3	16.9 (1.6)	31.9 (1.0)	32.5 (3.1)	7.1 (1.1)	18.3 (2.7)	27.6 (4.3)
M4	19.3 (1.7)	32.9 (1.1)	33.7 (2.4)	5.9 (0.2)	17.4 (1.3)	26.1 (2.2)
M5	12.6 (5.0)	21.8 (1.5)	19.4 (2.8)	5.9 (1.1)	14.4 (2.9)	20.6 (3.9)
M6	14.1 (5.2)	20.1 (3.0)	14.0 (4.0)	4.6 (0.5)	11.1 (1.1)	16.9 (1.3)
M7	18.6 (2.1)	29.2 (1.5)	26.2 (2.2)	6.5 (0.6)	16.4 (1.5)	22.9 (1.7)
M8	17.1 (2.9)	26.4 (1.8)	23.3 (2.1)	5.2 (0.5)	12.9 (0.9)	20.7 (1.3)
M9	19.6 (3.4)	23.4 (2.2)	29.9 (6.1)	3.6 (0.5)	8.6 (1.5)	13.4 (3.2)
U1	27.2 (1.3)	37.7 (1.2)	47.9 (3.0)	11.2 (0.7)	26.4 (1.6)	34.9 (1.7)
U2	23.7 (2.8)	36.3 (1.1)	46.8 (1.6)	9.7 (0.6)	26.1 (1.7)	35.4 (2.6)
U3	26.0 (2.8)	36.9 (1.1)	49.1 (2.3)	8.6 (0.3)	24.8 (1.3)	34.8 (1.6)
U4	26.0 (2.6)	37.2 (0.7)	49.4 (1.3)	6.5 (0.4)	18.3 (1.6)	24.7 (1.5)
U5	32.3 (2.7)	36.5 (1.7)	45.7 (2.1)	8.8 (0.5)	25.4 (1.8)	33.6 (2.7)
U6	27.7 (2.7)	36.7 (1.0)	47.0 (1.3)	8.0 (0.5)	23.5 (1.0)	32.19 (1.2)

Supplementary Table S4. Output from ANOVA for phenology and height variation in the three pine species. Degrees of freedom: d.f ; mean sum of squares: m.s.; F: F value; p: significance value.

**Source of variation**	**Bud set 1st year (2010)**				**Bud burst 2nd year (2011)**				**Bud burst 3rd year (2012)**			
	**d.f.**	**m.s.**	**F**	**p**	**d.f.**	**m.s.**	**F**	**p**	**d.f.**	**m.s.**	**F**	**p**
***Pinus sylvestris*_mainland**												
Population	6	14854	44.00	<.0001	6	2051	23.07	<.0001	6	6404	20.375	<.0001
Families within population	26	337.6	2.272	0.041	26	88.92	0.837	0.667	26	314.3	0.446	0.969
Block	17	148.6			17	106.2			17	705.4		
Residual	330	80.81			340	34.71			335	125.8		
***Pinus sylvestris*_Scotland**												
Population	5	354.3	1.502	0.230	5	204	2.386	0.071	5	380.1	1.183	0.349
Families within population	22	235.9	2.679	0.021	22	85.47	1.444	0.222	22	321.2	0.265	0.998
Block	17	88.06			17	59.18			17	1211		
Residual	314	71.8			319	28.37			316	172.3		
Pinus mugo												
Population	8	1030	3.638	0.005	8	743.6	7.491	<.0001	8	2243	7.855	<.0001
Families within population	29	283.1	1.279	0.302	28	99.26	1.086	0.440	28	285.5	0.461	0.967
Block	17	221.3			17	91.4			17	619.4		
Residual	338	121.5			343	50.28			340	118.3		
Pinus uncinata												
Population	5	565.4	1.199	0.340	5	24.67	0.258	0.931	5	162	0.867	0.518
Families within population	24	471.6	6.661	<.0001	24	95.55	2.401	0.034	24	186.9	0.225	1.000
Block	17	70.8			17	39.79			17	831.1		
Residual	416	105.1			419	21.77			416	90.06		
	**Height 2nd year (2011)**				**Height 3rd year (2012)**				**Height 4th year (2013)**			
	**d.f.**	**m.s.**	**F**	**p**	**d.f.**	**m.s.**	**F**	**p**	**d.f.**	**m.s.**	**F**	**p**
***Pinus sylvestris*_mainland**												
Population	6	1494	9.831	<.0001	6	7340	22.58	<.0001	6	8214	16.922	<.0001
Families within population	26	151.9	2.666	0.020	26	325.1	0.467	0.961	26	485.4	0.670	0.826
Block	17	56.99			17	696.8			17	724.6		
Residual	340	22			337	111.9			329	165.2		
***Pinus sylvestris*_Scotland**												
Population	5	190.9	2.142	0.098	5	702.3	2.408	0.069	5	904.3	2.611	0.053
Families within population	22	89.13	2.357	0.038	22	291.7	0.373	0.984	22	346.4	0.348	0.989

Block	17	37.82			17	781.2			17	994.7		
Residual	320	20.87			318	103.7			311	156.6		
Pinus mugo												
Population	8	44.09	2.214	0.056	8	469.9	3.740	0.004	8	851.6	3.137	0.011
Families within population	29	19.91	4.043	0.002	29	125.6	0.782	0.728	29	271.5	1.004	0.512
Block	17	4.924			17	160.6			17	270.4		
Residual	344	4.344			339	28.3			333	60.56		
Pinus uncinata												
Population	5	194.3	9.882	<.0001	5	728	5.033	0.003	5	1331	5.376	0.002
Families within population	24	19.67	2.660	0.021	24	144.6	0.695	0.797	24	247.6	0.860	0.640
Block	17	7.393			17	208			17	287.8		
Residual	417	4.979			417	38.36			410	75.67		

Supplementary Table S5. Variance components for phenology and growth traits based on a REML approach**.** For this purpose, each component (population, family with population, and block) was fitted as a random effect in the package lme4**.**

			% of total variance			
**Trait**	**Species**	**Mean**	**Population**	**Family**	**Block**	**Residual**
**Bud set 1st year (2010)**	*P. sylvestris* (mainland)	37.58 (6.89)	75.28	5.11	0.80	18.80
	P. sylvestris (Scotland)	36.21 (1.08)	2.77	14.77	1.20	81.25
	P. mugo	19.31 (1.58)	7.26	11.08	3.77	77.89
	P. uncinata	27.20 (1.59)	1.23	18.79	0.00	79.97
**Bud burst 2nd year (2011)**	*P. sylvestris* (mainland)	32.74 (2.67)	51.73	5.30	4.20	38.77
	P. sylvestris (Scotland)	35.67 (0.75)	3.79	12.59	4.37	79.25
	P. mugo	26.95 (2.83)	25.43	5.36	2.95	66.26
	P. uncinata	5.77 (0.36)	0.00	15.36	2.66	81.99
**Bud burst 3rd year (**2012)	*P. sylvestris* (mainland)	33.07 (4.51)	42.34	5.47	9.55	42.64
	P. sylvestris (Scotland)	42.12 (2.14)	2.34	3.84	21.13	72.69
	P. mugo	15.40 (1.21)	25.07	7.41	10.60	56.92
	P. uncinata	23.07 (1.57)	0.00	4.41	22.37	73.22
**Height 2nd year (2011)**	*P. sylvestris* (mainland)	13.74 (1.99)	42.26	16.84	2.80	38.10
	P. sylvestris (Scotland)	13.86 (0.81)	6.77	19.14	3.04	71.06
	P. mugo	5.76 (0.36)	10.36	21.68	0.77	67.19
	P. uncinata	8.83 (0.65)	27.21	11.14	1.19	60.47
**Height 3rd year (2012)**	*P. sylvestris* (mainland)	35.64 (4.46)	42.40	8.06	10.24	39.30
	P. sylvestris (Scotland)	34.55 (1.95)	4.30	9.90	20.18	65.63
	P. mugo	15.42 (1.2)	13.38	17.28	12.50	56.85
	P. uncinata	24.04 (1.35)	11.75	11.88	11.08	65.29
**Height 4th year (2013)**	*P. sylvestris* (mainland)	49.76 (5.03)	45.06	9.00	5.75	40.19
	P. sylvestris (Scotland)	47.69 (2.19)	3.92	7.41	17.95	70.72
	P. mugo	23.09 (1.56)	9.48	19.59	10.43	60.51
	P. uncinata	32.57 (1.76)	11.43	10.95	7.59	70.03

Supplementary Table S6. Covariation between selected trait pairs (A-D). Models with a covariate were compared with a corresponding null model; the best (in terms of AICc) is presented in each case. ΔAICc values represent the difference in AICc between the null model (no fixed effects) and the best (a value of zero indicates that the null model was the best); Akaike Weight represents the probability of that model being the best out of the set considered; df –degrees of freedom.

**A. Trait**	**Species**	**Intercept (SE)**	**Bud set 1st Year (2010) (SE)**	**df**	**ΔAICc**	**Akaike Weight**
**Bud burst 2nd year (2011)**	*P. sylvestris* (mainland)	34.82 (3.24)	-0.06 (0.03)	6	0.69	0.59
	P. sylvestris (Scotland)	39.36 (1.42)	-0.10 (0.03)	6	6.43	0.96
	P. mugo	31.71 (1.86)	-0.24 (0.03)	6	52.70	1.00
	P. uncinata	41.14 (0.69)	-0.16 (0.02)	6	53.79	1.00
**B. Trait**	**Species**	**Intercept (SE)**	**Bud set 1st Year (2010) (SE)**	**df**	**ΔAICc**	**Akaike Weight**
**Height 2nd year (2011)**	*P. sylvestris* (mainland)	14.01 (1.93)	-	5	0.00	0.54
	P. sylvestris (Scotland)	20.99 (1.20)	-0.20 (0.03)	6	47.5	1.00
	P. mugo	6.30 (0.41)	-0.03 (0.01)	6	5.70	0.95
	P. uncinata	8.85 (0.65)	-	5	0.00	0.73
**C. Trait**	**Species**	**Intercept (SE)**	**Bud burst 2nd Year (2011) (SE)**	**df**	**ΔAICc**	**Akaike Weight**
**Height 2nd year (2011)**	*P. sylvestris* (mainland)	13.96 (1.93)	-	5	0.00	0.57
	P. sylvestris (Scotland)	9.90 (1.81)	0.11 (0.05)	6	3.98	0.88
	P. mugo	4.20 (0.51)	0.06 (0.01)	6	13.79	1.00
	P. uncinata	6.92 (1.06)	0.05 (0.02)	6	3.04	0.82
**D. Trait**	**Species**	**Intercept (SE)**	**Bud burst 3rd Year (2012) (SE)**	**df**	**ΔAICc**	**Akaike Weight**
**Height 3rd year (2012)**	*P. sylvestris* (mainland)	29.62 (4.32)	0.18 (0.05)	6	11.86	1.00
	P. sylvestris (Scotland)	29.98 (2.61)	0.11 (0.04)	6	4.64	0.91
	P. mugo	14.05 (1.34)	0.05 (0.02)	6	2.24	0.75
	P. uncinata	24.07 (1.33)	-	5	0.00	0.70

Appendix 1. Evaluating Effects of Covariates in R.

```
//load relevant packages

library(lme4)

library(arm)

library(MuMIn)

//fit 'global' model for given species/trait using maximum likelihood

global_model<-lmer(Trait~LAT+LONG+(1|POP/FAM)+(1|BLOCK), REML=F, na.action="na.fail", data=data_set)

//standardise input variables

global_model_std<-standardize(global_model, standardize.y = FALSE)

//compare model subsets via AICc

dredge(global_model_std)

//Re-specify 'best' model by AICc using restricted maximum likelihood to obtain coefficient estimates e.g.

best_model<-lmer(Trait~LAT+(1|POP/FAM)+(1|BLOCK), REML=T, na.action="na.fail", data=data_set)
```

Supplementary Figure S1. Characteristics of species and populations in terms of altitude and latitude.

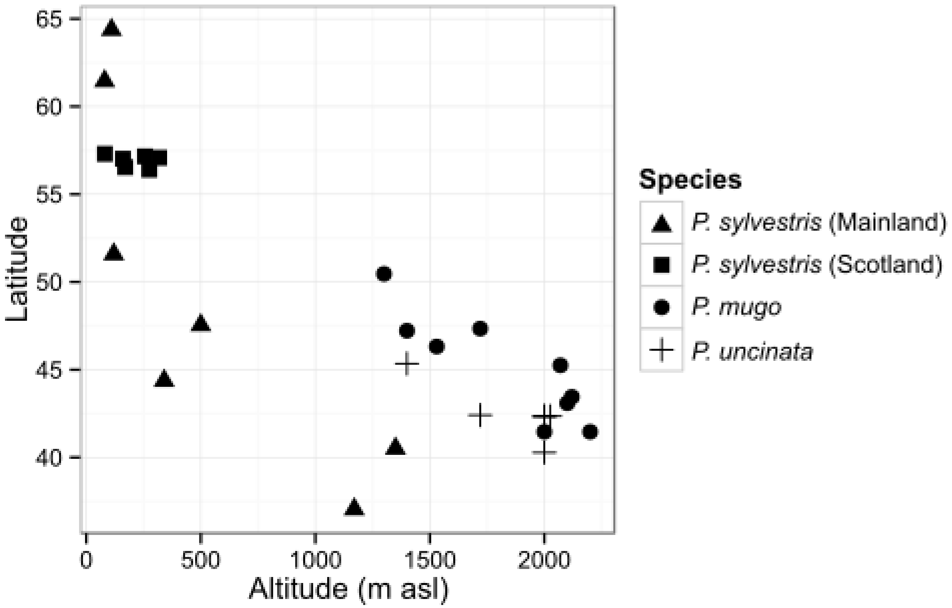

Supplement: Supplementary file 1 [file ECE3-8-655-s001.pdf]
